# Molten‐Volcanic‐Ash‐Phobic Thermal Barrier Coating based on Biomimetic Structure

**DOI:** 10.1002/advs.202205156

**Published:** 2023-02-02

**Authors:** Yiqian Guo, Wenjia Song, Lei Guo, Xinxin Li, Wenting He, Xudong Yan, Donald B. Dingwell, Hongbo Guo

**Affiliations:** ^1^ School of Materials Science and Engineering Beihang University Xueyuan Road 37 Beijing 100191 China; ^2^ Tianmushan Laboratory Xixi Octagon City, Yuhang District Hangzhou 310023 China; ^3^ School of Materials Science and Engineering Tianjin University Weijin Road 92 Tianjin 300072 China; ^4^ School of Mechanical Engineering and Automation Beihang University Xueyuan Road 37 Beijing 100191 China; ^5^ Department of Earth and Environmental Sciences Ludwig‐Maximilians‐Universität München Theresienstrasse 41 80333 Munich Germany

**Keywords:** biomimetic, hierarchical structure, thermal barrier coating, volcanic ash, wetting

## Abstract

Volcanic ash is a major threat to aviation safety. The softening/melting temperatures of volcanic ash lie far below typical aero‐engine operating temperatures. Thus, molten ash can accelerate the failure of thermal barrier coatings (TBCs). Here, inspired by natural superhydrophobic surfaces (e.g., the lotus leaf), a molten‐volcanic‐ash‐phobic TBC, which provides a large possibility to eliminate molten ash issues of TBCs, is developed. A hierarchically structured surface is first prepared on a (Gd_0.9_Yb_0.1_)_2_Zr_2_O_7_ (GYbZ) pellet by ultrafast laser direct writing technology, aiming to confirm the feasibility of the biomimetic microstructure to repel molten volcanic ash wetting. Then biomimetic‐structured GYbZ TBCs are successfully fabricated using plasma spray physical vapor deposition, which reveals “silicate” phobicity at high temperatures. The exciting molten‐volcanic‐ash‐phobic attribute of the designed surfaces is attributed to the lotus‐leaf‐like dual‐scale microstructure, emulating in particular the existence of nanoparticles. These findings may be an important step toward the development of next‐generation aviation engines with greatly reduced vulnerability to environmental siliceous debris.

## Graphical abstract

The laser‐ablated (Gd_0.9_Yb_0.1_)_2_Zr_2_O_7_ (GYbZ) pellet and plasma spray physical vapor deposition GYbZ coating demonstrate volcanic ash phobicity at 1200 °C. The results highlight that the biomimetic hierachical structure consisting of microconical papillae with aspect ratio of 1.10 ± 0.15 and regularly distributed nanoparticles makes great contributions to silicate repellence.

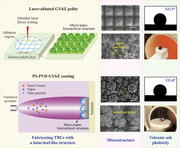

## 1 Introduction

In the past 30 years, over 100 commercial aircrafts have inadvertently traversed volcanic ash clouds with variably deleterious results.^[^ ^1^, ^2^ ^]^ A notable example of the effects of volcanic ash on air traffic was observed during the Eyjafjallajökull volcano eruption in 2010, which resulted in the most extensive air‐traffic shut down since the second world war and major financial repercussions (losses of up to €1.7 Billion).^[^ ^3^ ^]^ When volcanic ash‐laden air is ingested into aviation engines, volcanic ash particles liquify in the combustion chamber adhering to the hot section components (e.g., nozzle guide vanes and high‐pressure turbine blades); whereupon the molten silicate deposit, often approximated as a CaO‐Al_2_O_3_‐MgO‐SiO_2_ (CMAS) melt, actively clogs engine parts and attacks the protective thermal barrier coatings (TBCs) on turbine components.^[^ ^4^, ^5^ ^]^ TBCs, consisting of a ceramic topcoat and a metallic bond‐coat, are designed to protect the underlying superalloy components against corrosive gases at extreme temperatures.^[^ ^6^ ^]^ The top ceramic coating made of ZrO_2_ stabilized by 6–8 wt% Y_2_O_3_ (commonly referred to as YSZ) exhibits a tetragonal prime phase.^[^ ^7^ ^]^ In aero‐engines, there are two main kinds of commercial TBCs, one of which found on rotating parts (turbine blades, vanes) produced by electron‐beam physical vapor deposition (EB‐PVD), and the other of which is found on stationary parts (stator vanes, combustors, shrouds, after‐burners) produced by air plasma spray (APS). Both have been reported to have CMAS issues. Since the 1980s YSZ has been the standard material for TBC applications, and represented a major step forward for the development of TBCs.^[^ ^8^, ^9^ ^]^

TBCs experience a highly “violent” environment in a gas‐turbine engine, e.g., the TBCs on vanes face 10 atm pressure and gases at velocities of ≈300 m s^−1^ for hundreds of hours, and those on turbine blades undergo 20 000 r min^−1^ during the engine operation.^[^ ^10^, ^11^ ^]^ For a time, erosion resulting from the ingested particles had become a great thereat to the safety of TBCs, and was once thought it was a key mechanism for coating failure.^[^ ^12^, ^13^, ^14^ ^]^ As the drive to more efficient engines continues, the engine combustor temperature, turbine entry temperature, and TBC surface temperature become higher and higher, which could be far higher than the melting points of the CMAS. As a result, the ingested particles exhibit a liquid or a semi molten state before crashing onto TBC surfaces. This causes less damage to TBCs as compared to the solid particles. Furthermore, the rapid development of the TBCs fabrication technology, especially the emergence of plasma spray‐physical vapor deposition (PS‐PVD) technology, has largely improved the erosion resistance of TBCs.^[^ ^15^, ^16^ ^]^ Hence, coating erosion caused by the ingested particles is no longer an issue worth consideration. However, the molten CMAS adhered onto the TBC surfaces causes another thereat to TBCs. Once CMAS wets and spreads across the surface of TBCs (APS or EB‐PVD), YSZ is under chemical attack and the dissolution of YSZ grains into the CMAS melt yields precipitation of Y‐depleted ZrO_2_, accompanied by the deleterious volumetric effects of its tetragonal/monoclinic transition, leading to degradation of the TBC.^[^ ^17^, ^18^, ^19^, ^20^ ^]^ Therefore, the avoidance of CMAS wetting and corrosion of TBCs has become a research priority.

Surface microstructure is a key parameter controlling the melt wettability. Two types of typical coating microstructures, including APS TBCs with lamellar structure and EB‐PVD TBCs with columnar structure, exhibit topographic effects, which have limited resistance to molten CMAS wetting and spreading.^[^ ^21^ ^]^ Therefore, although many efforts have been made to mitigate CMAS corrosion on TBCs, such as exploring alternative coating materials for YSZ, the efficiency is not much satisfactory. For example, Gd_2_Zr_2_O_7_ (GZO) has been considered as a promising candidate material for TBC applications mainly due to its good resistance to CMAS corrosion. At high temperatures, GZO rapidly reacts with the melt to form a highly stable apatite‐structure silicate combined with a fluorite‐structure phase, which constitutes an inert crystalline layer on the surface.^[^ ^22^ ^]^ Due to the latter further penetration of the melt is suppressed, and the inner regions remain stably isolated from molten CMAS attack. Recently, a Yb_2_O_3_‐doped GZO TBC with an optimal composition of (Gd_0.9_Yb_0.1_)_2_Zr_2_O_7_ (GYbZ) attracted more attention as it possesses ultralow thermal conductivity at temperatures up to 1600 °C, further enhanced resistance to CMAS penetration and a much longer thermal cycling lifetime than GZO and YSZ TBCs.^[^ ^23^, ^24^, ^25^ ^]^ However, these TBCs produced by APS and EB‐PVD still suffer from molten CMAS corrosion, forming an ≈10 µm crystalline reaction layer and melt‐penetrated regions tens of microns thick (Figure S1, Supporting Information). In a word, melt adhering to the TBC surface still undoubtedly damages the coating. Hence, the ideal solution to CMAS attack issues is in developing TBCs that prevent the wetting of the silicate melt on the coating surface in the first place.

Over geologic time species have through evolution explored a vast range of properties. A well‐known example is the extraordinary superhydrophobic nature of the lotus leaf.^[^ ^26^, ^27^ ^]^ To prevent the wetting of the silicate melt on TBC surfaces, it is expected that the melt adheres to the coating surfaces like water on the lotus leaf. As mentioned above, TBCs undergo high‐velocity gas or high‐speed rotation during the engine operation; as a result, the adhered CMAS melt on the TBCs can be blown away in a matter of minutes. It is known that the superhydrophobic behavior of the lotus leaf is closely related to the combined effects of the hierarchically structured surface (i.e., the randomly distributed papillae epidermal cells of microscale covered by fine hairs of nanometer‐scale) (Figure S2, Supporting Information) and leaf surface chemistry (i.e., epicuticular waxes on the top of these papillose cells).^[^ ^28^, ^29^ ^]^ Inspired by that, many attempts have been made to create superhydrophobic surfaces exhibiting high contact angles (≥150°) for a wide application of directional droplet transport, anti‐icing, water harvesting, and other related fields.^[^ ^30^, ^31^ ^]^ Such a biomimetic microstructure can also be introduced into the TBCs field to improve the coatings CMAS resistance at high temperatures. Central challenges are: how to fabricate such microstructures on TBCs; whether such microstructure can indeed resist molten CMAS wetting at high temperature; what coating fabrication technologies exist to generate such microstructures.

**Figure 1:**
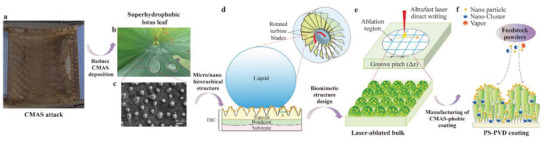
Schematic illustration of CMAS mitigation strategy and fabrication of micro‐nanostructured CMAS‐phobic TBCs.

To respond to these challenges, a schematic illustration for designing a biomimetic‐structured TBC has been first drawn, as shown in **Figure** **1**. Figure 1a–c provides a potential method for alleviating CMAS attack to TBCs, which is inspired by the lotus leaf that is highly resistant to water wetting. If the TBC surfaces have a biomimetic microstructure like the lotus leaf, together with the high‐velocity gas or high‐speed rotation, the molten CMAS cannot be adhered to and stay on to the TBCs, as illustrated in Figure 1d. To evaluate the ability of this biomimetic microstructure repelling molten CMAS wetting, a hierarchically structured GYbZ pellet surface was first prepared using an ultrafast laser direct writing strategy, as shown in Figure 1e. After ensuring that the biomimetic microstructure (hierarchically structured surface) can indeed resist molten CMAS wetting at high temperature, we attempt to construct this type of biomimetic microstructure on GYbZ TBC surfaces using state‐of‐the‐art PS‐PVD technology, as shown in Figure 1f. Finally, a molten‐volcanic‐ash‐phobic TBC was successfully obtained, which has a contact angle with the melt of ≈121.4° at high temperature (1200 °C). Due to the excellent erosion resistance of PS‐PVD TBCs, the fabricated micro‐ and nanohierarchically structured coatings are expected to resist against molten silicate adhesion and wetting in the gas‐turbine engine environment.

## 2 Results and Discussions

### 2.1 Fabrication of Hierarchical Structure on GYbZ Surfaces via Ultrafast Laser Direct Writing

**Figure 2:**
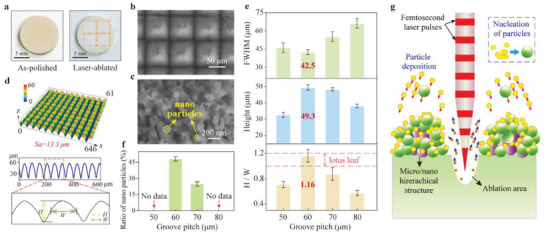
a) Optical images of as‐polished (left) and laser‐ablated (right) GYbZ bulks. The orange square shows the laser‐ablated area. b) SEM image of laser‐ablated GYbZ bulk with Δ*z* = 60 µm. c) High magnification showing nanoparticles in laser‐ablated area. d) The 3D surface topography and corresponding 2D profile of laser‐ablated GYbZ bulk with Δ*z* = 60 µm. The surface roughness is demonstrated by the Arithmetical mean height (*S*_a_). e) Characteristic geometrical parameters, FWHM, height, and H/W of laser‐ablated GYbZ bulks with different groove pitches. f) The ratio of nanoparticles of laser‐ablated GYbZ bulks with different groove pitches. g) Schematic of femtosecond laser ablation and deposition of nanoparticles.

The macroscopic morphologies of GYbZ pellets obtained before and after femtosecond laser treatments are compared in **Figure** **2**a. Laser ablation is accompanied by a change in pellet color from light yellow to white. It can be seen in Figure 2a that material has been removed along the laser scanning path. Scanning electron microscopy (SEM) observation of the laser‐ablated pellet surface reveals a characteristic microgrid structure with numerous microconical papillae, each of which is decorated with spherical nanoparticles (Figure 2b,c). This microstructure resembles that of the lotus leaf. The 3D surface topography is presented in Figure 2d, where the microconical papillary structure is characterized using confocal laser scanning microscopy (CLSM) in terms of the parameters of FWHM (full‐width at half‐maximum), height, and *H*/*W* (height/FWHM). The mean aspect ratio *H/W* of the conical papillae is 1.16 ± 0.10 (Figure 2e), similar to that of the lotus leaf (≈1.12 ± 0.10). Additionally, the nanoparticles on conical papillae of both our laser‐ablated pellet and the lotus leaf exhibit similar particle sizes < 200 nm and account for ≈50% of the total area (Figure 2f). GYbZ remained phase stable during the preparation of this microstructure (Figure S3, Supporting Information). It can be therefore concluded that a hierarchical microstructure has been successfully prepared here by an ultrafast laser direct writing technology.

The mechanism for the formation of the hierarchical microstructure consisting of microconical papillae and nanoparticles on a GYbZ pellet is elucidated in Figure 2g. When a high‐energy laser beam strikes the surface of a dielectric material, it induces multiphoton absorption and energy transfer from photons to free electrons. This further promotes the generation of a plasma cloud composed of high‐energy particles such as atoms, clusters, and nanoparticles. The high‐energy electrons then collide with bound electrons, resulting in more ionization‐induced free electrons and lead to a so‐called “Coulomb explosion.”^[^ ^32^, ^33^, ^34^ ^]^ Some of the products of Coulomb explosion are forced away from the ablation area, and dissipate in air due to the initial kinetic energy. This material is that which is removed through laser ablation. Other products fall back onto the surface via gravity and air pressure effects, yielding deposition of nanoparticles. Repeated laser scanning continuously deepens the ablation area, producing a regular microconical array structure as shown in Figure 2d.

Due to the Gaussian distribution of the laser beam energy, the energy absorbed in the GYbZ pellet decreases with depth, yielding grooves of V‐shaped geometry (Figure 2d). The groove pitch (Δ*z*), which has a significant effect on the width and area of the microprotrusion, as well as the formation and distribution of nanoparticles, is ≈60 µm in Figure 2b. Laser‐ablated GYbZ pellets with other Δ*z* values were further fabricated. Increasing the groove pitch from 50 to 80 µm yields increased width and area of the microprotrusions, while the surface roughness of the laser‐ablated pellet shows no apparent change (Figure S4a, Supporting Information). SEM observations indicate that the microprotrusions are evenly distributed on the pellet surface regardless of groove pitch, whereas higher magnification reveals that their detailed microstructure is affected by the groove pitch (Figure S4b, Supporting Information). At Δ*z* = 50 µm, the protrusions are completely covered by rough microaggregates, on which some nanoscale pores are irregularly distributed. Increasing the groove pitch leads to nanoparticle formation on the microconical papillae. At Δ*z* = 60 and 70 µm, the nanoparticle areas account for ≈48% and ≈28% of the total areas, respectively. It should be noted that nanoparticles on the microconical papillae disappear with larger groove pitch. At Δ*z* = 80 µm, no nanoparticles are observed and microripples are present instead.

### 2.2 Feasibility of the Hierarchical Microstructure to Repel Molten Volcanic Ash Wetting

**Figure 3:**
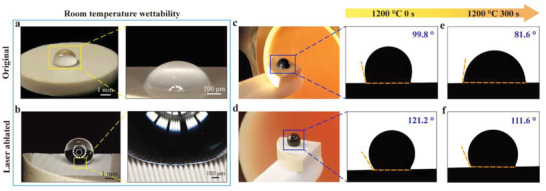
Wetting properties of original and laser‐ablated GYbZ bulks. a) Hydrophilicity of original bulk and b) superhydrophobicity of femtosecond laser ablated bulk (*∆z* = 60 µm). Magnifications of interface between water droplet and solid surface (right). Variation of wettability at 1200 °C with dwelling time increasing: c,e) original GYbZ bulk and d,f) laser‐ablated GYbZ bulk (*∆z* = 60 µm). Actual photographs (left) and contact angles (right) of molten silicate droplet on c) original and d) laser‐ablated GYbZ bulk in furnace after heating to 1200 °C. Contact angles of molten silicate on e) original and f) laser‐ablated GYbZ bulk attacked with dwelling time of 300 s at 1200 °C.

Static sessile drop contact angle measurements have been conducted on the laser‐ablated GYbZ pellets to test for hydrophobicity. The as‐polished pellet exhibits a water contact angle of 86°, while the laser‐ablated GYbZ pellets and especially that with a hierarchical microstructure consisting of microconical papillae and nanoparticles (Figure S5, Supporting Information) (Δ*z* = 60 µm), exhibit a water contact angle of 151 ± 2° (**Figure** **3**b). Thus, our lotus‐leaf‐inspired laser‐ablated hierarchical microstructure possesses a superhydrophobic character. Next, we investigate the possibility of CMAS‐phobicity at high temperature.

Due to its lower temperature liquification, volcanic ash represents an even greater threat to turbine ingestion than other environmental contaminants such as sand and dust. Here, we investigate a volcanic ash sample collected from the Eyjafjallajökull eruption in Iceland on April 15th, 2010,^[^ ^35^ ^]^ and examine the capability of our fabricated hierarchical microstructure to repel molten silicate wetting. The physicochemical properties of our Eyjafjallajökull volcanic ash were systematically parameterized (Figure S6, Supporting Information). Its chemical composition was analyzed using X‐ray fluorescence (XRF). The silica concentration in this trachyandesite is ≈60%. This sample has been observed to start to liquify at ≈1160 °C, consistent with previous studies on Eyjafjallajökull volcanic ash sample.^[^ ^36^, ^37^ ^]^

The morphological characteristic of volcanic ash particles was obtained using SEM and laser diffraction methods. The original volcanic ash fragments are of irregular shape with circularities from 0.4 to 1.0, with 70% of particles between 0.7 and 0.9 (see Figure S6b,c, Supporting Information). The particle size distribution of volcanic ash samples is expressed as the *D*_10_, *D*_50_, and *D*_90_ fractions of the cumulative volume distribution, the characteristic values of which constrain the smallest particle sizes. The *D*_90_ of volcanic ash originally collected is ≈44.5 µm, which is much coarser than the specifications for powder compaction preparation (<20 µm). To meet the sample preparation requirements for high‐temperature experiments, the originally collected particles are ground. The shape and size of the ground particles are more uniform, approximately spherical (close to 1.0) and finer (*D*_10_ = 1.11 µm, *D*_50_ = 4.07 µm, *D*_90_ = 15.90 µm). The melting behavior of the ground volcanic ash was determined using a differential scanning calorimeter (DSC; Figure S6e, Supporting Information). The initial melting temperature of the volcanic ash is found to be ≈1164 °C. Thus, the target temperature for wetting experiments was set at 1200 °C to stimulate the melting behavior of volcanic ash in a hot turbine, under the condition of molten volcanic ash.

A series of experiments were conducted on the molten CMAS wetting behavior on laser‐ablated GYbZ pellets with different ablation parameters (i.e., Δ*z* = 50, 60, 70, and 80 µm). Volcanic ash samples (with grain size of <20 µm) were prepared into pressed cylinders of 2 mm in height and 2 mm in diameter, which were then placed on GYbZ pellets and heated to 1200 °C at a rate of 5 °C min^−1^ with a dwell time of 300 s, in air. The sessile drop method was used to measure the contact angle between the melt and pellets and record the geometrical evolution of cylindrical CMAS sample such as height, baseline length, *R*, and radius with respect to time and temperature (Figure 5b). As presented in Figure S7 in the Supporting Information, as was observed for low temperature measurements with water, here, the laser‐ablated pellets exhibited larger contact angles with molten CMAS than the original pellet.

In particular, laser‐ablated pellet with hierarchical microstructure (Δ*z* = 60 µm, Figure 3) exhibits contact angles up to ≈121.2° at 1200 °C, about 21° higher than that of originally synthesized/untreated pellets (≈99.8°). It can be concluded that GYbZ with hierarchical microstructure (Δ*z* = 60 µm) can effectively resist CMAS wetting at high temperature. After 300 s at 1200 °C, the laser‐ablated pellet maintains good antiwetting performance, with a contact angle as high as 111.6° indicating excellent thermal stability of the fabricated hierarchical microstructure.

### 2.3 Construction of a Micro‐/Nanoscale Hierarchically Structured TBC via PS‐PVD

A gap remains for the practical application of these results to the field of TBC fabrication. Turbine blades, vanes, and other engine hot parts that require TBCs for thermal protection usually have complex profiles. As a result, using the ultrafast laser direct writing technology to efficiently and precisely obtain the micro/nanohierarchical microstructures on the surface of TBCs still faces major engineering challenges.

**Figure 4:**
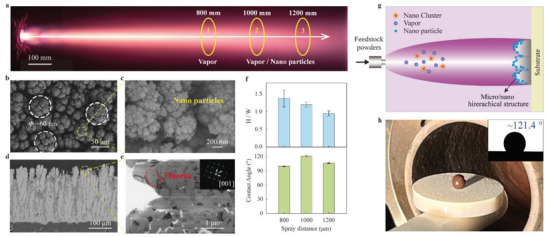
Wetting behavior of molten silicate on PS‐PVD GYbZ coating. a) Photograph of PS‐PVD plasma plume along with the axial direction. b) Surface morphology of PS‐PVD GYbZ coating sprayed at 1000 mm. c) Magnified nanoparticles on the surface of micropapilla in (b). d) Cross‐sectional SEM image of PS‐PVD GYbZ coating. e) Bright‐field TEM image of micropapillae encircled by yellow dash in (d). Electron diffraction pattern oriented along a [001]‐type zone axis confirming the presence of GYbZ fluorite phase within the dashed oval area (top right). f) Mean ratio of H/W (top) and contact angles of molten silicate on PS‐PVD coatings sprayed at different distance (bottom). g) Schematic of micro‐ and nanohierarchical structural coating performed by PS‐PVD. h) Photograph of silicate droplet of 2 mm onto the PS‐PVD coating at 1200 °C. Top right, contact angle of silicate droplet on the as‐sprayed coating.

As noted above, traditional TBCs are manufactured by EB‐PVD or APS. Unfortunately, both EB‐PVD and APS TBCs, with a columnar or lamellar structure, are highly susceptible to contamination and wetting by CMAS.^[^ ^15^ ^]^ Current state‐of‐the‐art techniques of PS‐PVD, especially those involving long plasma plumes, may provide a possibility to overcome this challenge.^[^ ^15^ ^]^ In contrast to the deposition processes of APS and EB‐PVD, agglomerated GYbZ powders in the PS‐PVD process were first heated, melted, and even evaporated within microseconds by a plasma plume with an extremely high temperature (≈2 × 10^4^ °C), then deposited onto the Al_2_O_3_ substrate in the form of vapor, liquid droplets, and nanoclusters (**Figure** **4**a). Architectures of these coating structures can be practically and precisely tailored by adjusting processing parameters, such as spray distance and substrate temperature.^[^ ^38^, ^39^ ^]^

The surface morphology of a GYbZ TBC produced by PS‐PVD at the spray distance of 1000 mm is shown in Figure 4a, where it is directly compared to those of GYbZ pellets after laser ablation with Δ*z* = 60 µm (see above) (Figure 4c). The PS‐PVD process generates surfaces dominated by hemispherically topped papillae (55–65 µm; aspect ratio 1.19 ± 0.07, Figure 4f) that are decorated by nanoparticles. The nanoparticle phase is revealed by transmission electron microscope (TEM) measurements to be a fluorite‐structure GYbZ phase <200 nm in size (Figure 4e), indicating that fabrication of the hierarchical microstructure by PS‐PVD does not precipitate any phase transformation and/or decomposition.

A mechanism for the formation of hierarchically structured TBCs consisting of microconical papillae and nanoparticles by PS‐PVD is illustrated in Figure 4g. During the PS‐PVD process, the agglomerated powders are transported into the center of plasma plume and deposited onto the preheated substrate positioned at a certain spraying distance. Some GYbZ particles are vaporized and deposited to form a columnar microstructure that exhibits conical papillae appearance from the surface view. Due to the presence of nanoparticles from feedstock powders or nanoclusters from condensation of vapor phase, some deposit phases are stacked onto the neighboring microcolumns to produce new microdendrites, while others are distributed regularly onto the conical papillae. Hierarchically structured TBCs are composed of microconical papillae and nanoparticles.

Due to the fact that the microstructure of GYbZ TBCs is greatly affected by the PS‐PVD process parameters, especially the spraying distance, fabrication of micro/nanohierarchical microstructures TBC with a lotus leaf‐like microstructure using this advanced spraying technology may be very complex. For example, at a short spraying distance of 800 mm (position 1 in Figure 4a), the formation of a coating is largely governed by nucleation and growth of nanoliquid phase after condensation of vapor phase in a manner similar to EB‐PVD coating, resulting in microcolumnar microstructure with aspect ratio of 1.37 ± 0.20 without decoration with nanoparticles (Figure S8a, Supporting Information). The aspect ratio clearly decreases as the distance is increased (Figure 4f, top). At the longest spraying distance of 1200 mm (position 3 in Figure 4a), most of unevaporated particles escape from the center of the plasma jet, such that the droplets from condensation of vapor phase become coarser, greatly reducing the propensity for the formation of nanoparticles on the conical papillae (in Figure S8d–f, Supporting Information).

To verify whether a GYbZ TBC produced with micro/nanohierarchical microstructures by PS‐PVD at a spaying distance of 1000 mm is able to resist CMAS wetting, a sessile drop method was used to examine its molten CMAS wettability at 1200 °C. The CMAS cylinder (2 mm in height and 2 mm in diameter, preparation method is same to that in Section 2.2) on such a surface evolves to a nearly spherical drop at 1200 °C (Figure 4h), indicating excellent resistance to CMAS wetting with an optimal contact angle of ≈121.4° (Figure 4f), compared with TBCs sprayed at 800 and 1200 mm. These results indicate that this TBC possesses a high ability to repel molten CMAS wetting when the TBC possesses the lotus‐leaf‐inspired closed *H/W* of conical papillae and sufficient nanoparticles. Thus, this strategy of designing a lotus leaf‐like microstructure to resist CMAS wetting and improve CMAS resistance of TBCs appears feasible. Importantly, this structured TBC has been successfully fabricated and is thus available for further development.

### 2.4 The Mechanisms of Hierarchically Structured TBCs Restricting Volcanic Ash Wetting

**Figure 5:**
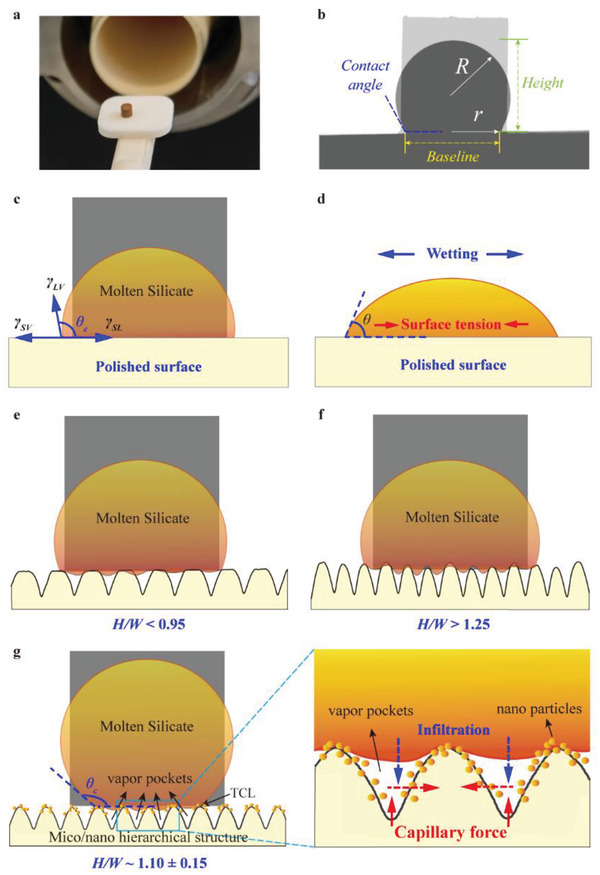
a) Actual photographs of sample assembly before heating. b) Schematic diagram of geometrical evolution involving height, baseline length, and contact angle of silicate deposits at representative states during wetting process. c) Equilibrium contact angle and d) spreading process of molten silicate on polished GYbZ surface. e) Schematic diagram of molten silicate on micropapillae (with aspect ratio < 0.95). f) Schematic diagram of molten silicate on micropapillae (with aspect ratio > 1.25). g) Schematic diagram of silicate phobicity on micro/nanohierarchical structures.

The potential mechanism responsible for CMAS resistance on the GYbZ substrates with hierarchical microstructure is outlined in **Figure** **5**. First, a cylindrical CMAS compact generally undergoes sequent sintering, melting, and wetting process, resulting in systematic geometrical evolution (Figure 5a,b) onto a flat substrate. The wetting process is characterized by the baseline between the sample‐substrate contact surface to quantitatively estimate the melt wetting behavior (Figure 5b). Our experimental results have shown that the microstructure of the coatings/pellet surface had substantial effects on the baseline length of molten CMAS with substrate (Figure S9a, Supporting Information). Hierarchically structured surfaces, regardless whether laser‐treated pellets or produced by PS‐PVD, enhance the repelling of molten CMAS wetting (whereby hierarchically structured surfaces of *H/W* = 1.16/1.19 ± 0.10 have the lowest value of the baseline length).

To further dissect the mechanism of the surface microstructure on the wettability of CMAS, several approaches have been proposed, such as those involving the Wenzel and the Cassie‐Baxter equations.^[^ ^40^ ^]^ First, the equilibrium contact angle *θ*_e_, which is determined on a polished GYbZ pellet is described by the Young's equation^[^ ^41^ ^]^

(1) $$\cos\;\theta_{e}=\frac{\gamma_{\mathrm{SV}\;-\;}\gamma_{\mathrm{SL}}}{\gamma_{\mathrm{LV}}}\;$$

In reality, the contact angle between the melt and a rough surface at the same experimental condition is referred as *θ*_c_, which could be described by Wenzel or Cassie models. The former assumes that the liquid wets the whole rough substrate, while the latter considers that the droplet partially wets the rough substrate due to the trapped air in the microstructures. In our experiments, the Cassie model might be more suitable for explanation of the mechanism for CMAS‐phobicity of our hierarchically structured surface because of the existence of trapped air in the superhydrophobicity experiments (Figure 3b). The contact angle (*θ*_c_) of which can be estimated as the Cassie model

(2) $$\cos\;\theta_{c}=f_{s}\;\;\left(\cos\theta_{e}+1\right)-1$$

where *f*_s_ is referred to the solid fraction of contact area between CMAS and substrate. In our experiments, the *θ*_e_ of CMAS drop on the surface with an ordinary microstructure is above 90°, the *θ*_c_ thus monotonically increases with decreasing *f*_s_. Therefore, the lower the value of *f*_s_ is, the higher the resistance to CMAS wettability the substrate possesses.

A morphology of microconical papillae (i.e., aspect ratio, *H/W*) and the formation of nanoparticles deposited on the surface of these papillae are the critical characteristic parameters associated with the value of the *f*_s_ for describing the surface microstructure of hierarchically structured samples. Based on our fabricated hierarchical microstructure of GYbZ substrates via laser‐ablated and PS‐PVD, the critical value of *H/W* is 1.10 ± 0.15, similar to lotus leaf values of 1.12 ± 0.10). Below this value, molten CMAS in contact with microconical papillae results in trapped vapor pockets beneath the liquid, forming a discontinuous triple‐phase contact line (Figure 5g). However, when the *H/W* is lower than 1.10 ± 0.15, the number of microconical papillae in contact with molten CMAS decreases, resulting in an increased solid fraction per unit area (i.e., the increase of *f*_s_), thereby higher *f*_s_ corresponding to a lower *θ*_c_ (Figure 5e). Conversely, when the *H/W* is higher than 1.10 ± 0.15, the increase of *f*_s_ renders the value of *θ*_c_ lower, as shown in Figure 5f. In addition, importantly, the entrapped air increases the surface energy, together with the nanoparticles, which could effectively reduce the adhesion and spreading force of the melt on the hierarchically structured surface and retard the melt wetting.

In this case, the inertia of the molten CMAS drops originating from the inner viscous force becomes lower, as the viscosity rapidly decreases with increasing temperature (Figure S10, Supporting Information), thus the resistance to the driving force for wetting derives principally from the unbalanced combined force of surface tension (Figure 5c,d). Consequently, the molten CMAS drop readily wets the polished GYbZ pellet. When the contact angle of CMAS reaches the maximum value, however, CMAS will inevitably spread onto the substrate. Thus, a fundamental understanding of the effect of microstructure on spreading dynamics of CMAS drop will be an essential input to the development of the new coating. In addition, in comparison of the spreading rate of cylindrical CMAS compacts on various surface microstructures, based on the evolution rate of the baseline length (i.e., *dr*/*dt*), the cylinder resting on hierarchical microstructures has lowest *dr*/*dt* values, further suggesting that this microstructure (microconical papillae and nanoparticles) also contributes to impeding the CMAS spreading.

## 3 Conclusions

In this study, the wetting and spreading process of molten silicate on laser‐ablated GYbZ pellets and PS‐PVD coatings varied by the parameters of laser or spraying processing, reveals the impact of surface microstructure. Taken together, the GYbZ pellet ablated by femtosecond laser with Δ*z* = 60 µm and PS‐PVD coating sprayed at 1000 mm demonstrate silicate phobicity at 1200 °C, the contact angles of molten silicate on both of which are more than 120°. This result highlights that the hierarchical structure consisting of microconical papillae with aspect ratio of 1.10 ± 0.15 and regularly distributed nanoparticles make great contributions to silicate repellence at high temperature. We expect that high interest in ultrafast laser direct writing technology and PS‐PVD technical process will lead to continued innovation in fabrication and structural optimization of TBCs against attack by various environmental siliceous debris. These results should inform the development of next‐generation land‐based and gas‐turbine engines for harsh service environments.

## 4 Experimental Section

### Preparation of GYbZ Pellets

(Gd_0.9_Yb_0.1_)_2_Zr_2_O_7_ (GYbZ) pellet samples were fabricated by solid state reaction method at 1600 °C for 24 h. GYbZ powder was synthesized by mechanically mixing the constituent oxides (Gd_2_O_3_, Yb_2_O_3_, ZrO_2_, all with the purity of higher than 99.99%) with absolute ethyl alcohol and alumina grinding media (for 8 h). After drying at 200 °C for 1.5 h, these powders were pressed into the pellets of 14 mm in diameter and 2 mm in thickness, and then densified by pressureless‐sintering at 1600 °C for 10 h in air. To eliminate the effect of original surface roughness, the sintered pellets were polished mechanically with P4000 silicon carbide paper (mirror‐polish) and then cleaned ultrasonically for 10 min, successively in acetone, ethanol, and deionized water, finally dried at 200 °C for 1.5 h prior to the following laser ablation (irradiation) processing.

### Ultrafast Laser Direct Writing Processing

In this study, an amplified Yb:KGW solid‐state laser system with 209 fs pulses of a central wavelength of 1030 nm with a repetition rate *f* of 100 kHz was used. The vertically polarized Gaussian laser beam (with an average power *P* of 5 W) was focused at normal incidence onto the sample surface using a galvanometric scanner with a telecentric f‐theta lens (Figure S11, Supporting Information, the schematic of experimental setup). The focused laser beam profile was observed by using a commercial M^2^ laser beam profiler (Duma, IR 10, Israel). The spatial distribution of the energy density in the focal spot was the Gaussian form (Figure S11b, Supporting information), where the Gaussian beam diameter *Ф* is 35 µm (responding to the position of 1/*e* ^2^ of the maximum optical intensity of beam). The optimized laser scanning speed of 15 mm s^−1^ and the scanning time of 10 were adopted.

These Gaussian‐shaped pulses were produced at a pulse energy *E*_p_ = 50 µJ, which was calculated by the following equation

(3) $$E_{p}=\frac{P}{f}\;$$

Femtosecond laser ablation happened on a short time after the collision between a pulse of a femtosecond laser and the material,^[^ ^42^ ^]^ where the feature size of ablation was determined by the peak laser fluence, *F*, which must exceed a certain ablation threshold value (*F*_th_) to cause an irreversible change in the surface. The peak laser fluence *F*_0_ was related to the measured incident laser pulse energy *E*_p_ by Equation (4)^[^ ^43^ ^]^

(4) $$F_{0}=\frac{8E_{P}}{\pi \omega_{0}^{2}}\;$$

With respect to this definition, the laser fluence *F*_0_ was set to 5.20 J cm^−2^ in the experiment, significantly above the ablation fluence for the used materials (e.g., 2.4 J cm^−2^ in ref. ^[^ ^44^ ^]^).

All the GYbZ pellets were polished first (left, Figure 1a), and then mounted on a high‐precision computerized *X*–*Y* translation stage, where a mechanical shutter was synchronized to the stage motion to provide a uniform exposure area. The line‐scanning approach in two mutually perpendicular directions was employed to manufacture a square array pattern of a 4.5 × 4.5 mm area with regular microgrid structures through material removal (right, Figure 1a). The interval distance between two successive laser beam tracks, as the one of the important process parameters, varied from 50 to 80 µm with each interval of ≈10 µm to achieve different groove pitches (Δ*z*) between V‐shaped microgrooves. To avoid excessive overlapping of parallel laser tracks, 50 µm was selected as the initial interval distance as the focal spot size on the surface of sample was around 35 µm. Following the laser irradiation process, all the samples were cleaned in ultrasonic baths of ethanol and deionized water treatment at room temperature for 10 min each time.

### Preparation of PS‐PVD GYbZ Coatings

The raw materials, Gd_2_O_3_ (99.99% purity) and ZrO_2_ (99.99% purity) and Yb_2_O_3_ (99.99% purity) in a precise stoichiometric ratio (Gd:Zr:Yb molar ratio of 9:10:1), along with deionized water and zirconia grinding balls were mixed for ball milling to get a homogenous mixing. The obtained slurry was dried, and then sintered at 1550 °C for 10 h. The feedstock powders were then manufactured by spray granulation reported in previous work.^[^ ^45^ ^]^ The GYbZ coating was deposited on Al_2_O_3_ substrate (purity ≥ 99.9%, Φ14 mm × 3 mm, Hesse Instruments, Germany) by PS‐PVD equipment (Medicoat AG, Switzerland) with an MC‐100 plasma torch, which allowed a maximum current of 3000 A and a peak‐power of 150 kW. The processing parameters for spraying are listed in Table S1 in the Supporting Information. Before powder injection, the substrates were preheated by plasma plume to a temperature of ≈900 °C that was detected by an IR pyrometer (Pyroview 380 Compact).

### Microstructure Characterization

The X‐ray diffraction pattern was carried out on X‐ray diffractometer (D/max‐2500, Rigaku, Japan) with Cu K*α* radiation (40 kV, 200 mA). Diffraction patterns were scanned from 10° to 70° with a scanning speed of 6° min^−1^. The effects of the laser irradiation on the surface morphology were investigated by means of CLSM (OLS 4100, Olympus) and SEM (Gemini 300, Zeiss). Profile scanning was also conducted (in the *x*, *y* direction of the surface) using CLSM over an area of 646 × 646 µm^2^. The surface roughness was evaluated using the roughness parameter, *S*_a_, which represents the average of the absolute height values of *Z*(*x*,*y*) in the measured area (*A*) and defined by $S_{a}=\frac{1}{A}\;\int \;\;\;\int_{A}\left|Z(x,y)\right|dxdy$.

### High Temperature Wettability Measurement

The volcanic ash collected from the 2010 eruption of Eyjafjallajökull volcano, Iceland, due to potential damage for aero‐engines, was used as the high temperature wettability measurement.^[^ ^35^ ^]^ The chemical compositions of volcanic ash were detected by XRF. The melting behavior of the volcanic ash was determined using a DSC coupled with thermo‐gravimetric analysis (TGA/DSC 3+, METTLER). The particle size distribution and geometrical characteristics (i.e., circularity) of ash samples were investigated by particle size analyzer (Bettersize 3000 Plus).

High temperature wettability measurement was carried out in a heating microscope (Hesse Instruments, Germany). The ash sample was first ground to the particles smaller than 20 µm, in accordance with the requirements of powder compacts preparation for wetting experiment in a heating microscope (CEN/TS 15 443 procedures). The milled ash was then compacted into cylindrical cores (2 mm × 2 mm) onto the GYbZ samples. The assembly of ash compact and GYbZ substrate was carefully positioned on the sample holder in the heating microscope and mitigated into the tube furnace. The sample together with ash compact was heated to 1200 °C at a rate of 5 °C min^−1^ with dwelling time of 1200 s in an atmosphere environment. Morphological changes (including height, baseline, and contact angle) of the compacts on heating were monitored in Heating Microscope EM301. Each sample was measured three times under the same conditions.

## Conflict of Interest

The authors declare no conflict of interest.

## Supporting information

Supporting Information

## Data Availability

The data that support the findings of this study are available from the corresponding author upon reasonable request.
