# Supplementary material for: Molten‐Volcanic‐Ash‐Phobic Thermal Barrier Coating based on Biomimetic Structure
Source: Adv Sci (Weinh). 2023 Feb 2;10(10):2205156. doi: 10.1002/advs.202205156 (PMC10074056; doi:10.1002/advs.202205156)
Supplement: Supplementary file 1 — Supporting Information [file ADVS-10-2205156-s001.pdf]

Supporting information

Biomimetic structured thermal barrier coating repelling molten volcanic ash wetting

Yiqian Guo, Wenjia Song, Lei Guo, Xinxin Li, Wenting He, Xudong Yan, Donald B. Dingwell, Hongbo Guo**

1. Figure S1 to S11

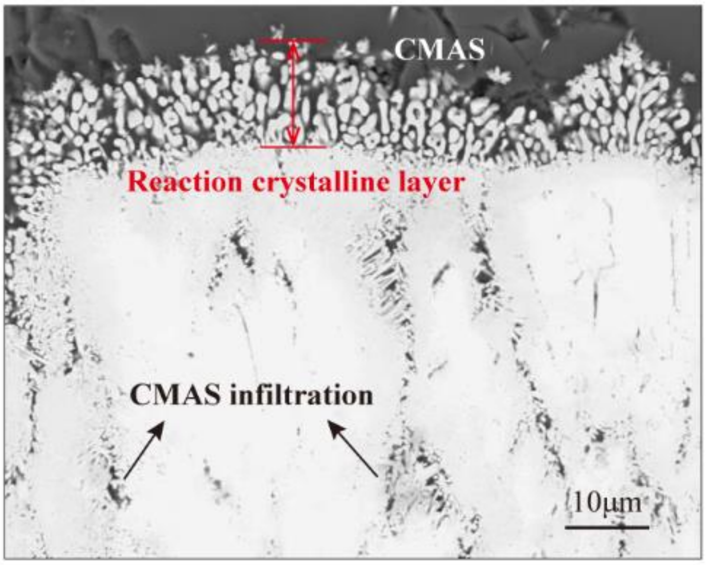

Figure S1. Cross-sectional SEM image of the EB-PVD GZO coating interacted with molten silicate.

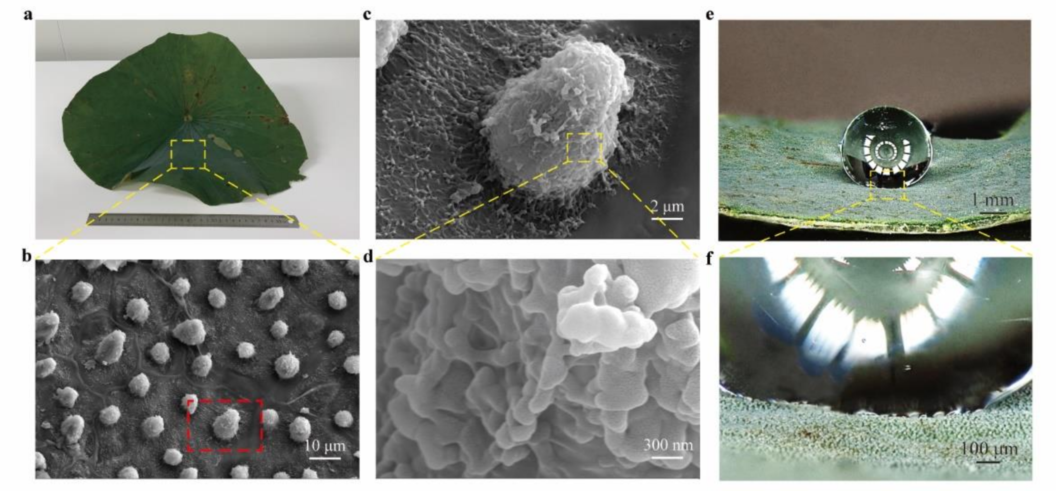

Figure S2. a) Photograph of Lotus leaf. b) SEM image of the lotus leaf surface comprising conical papillae. c) Higher magnification SEM image of a single papillose showing micro/nano-hierarchical structures. d) Higher magnification SEM image of nano particles in the region circled by yellow rectangle in (c). e) Superhydrophobicity on the surface of lotus leaf.

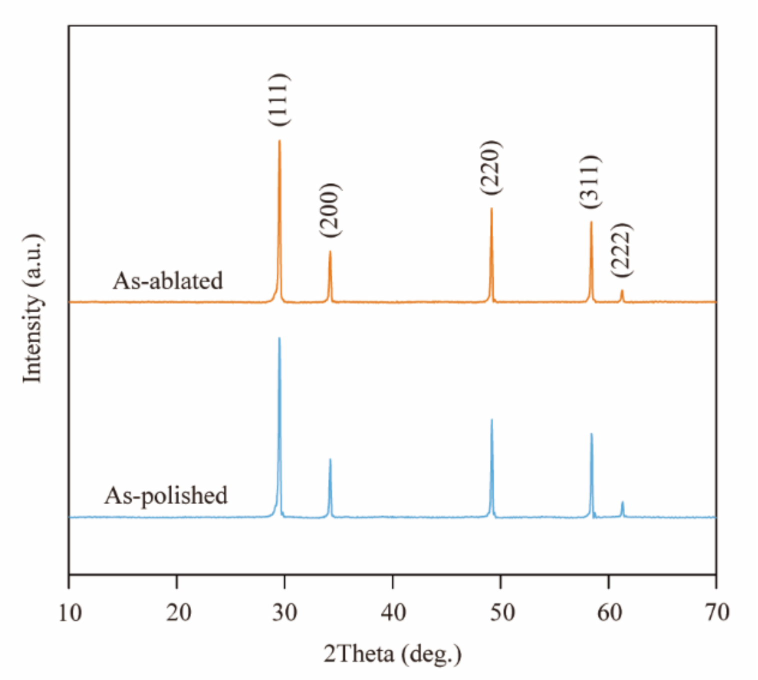

Figure S3. XRD patterns of as-polished (blue curve) and as-ablated (orange curve) GYbZ bulks.

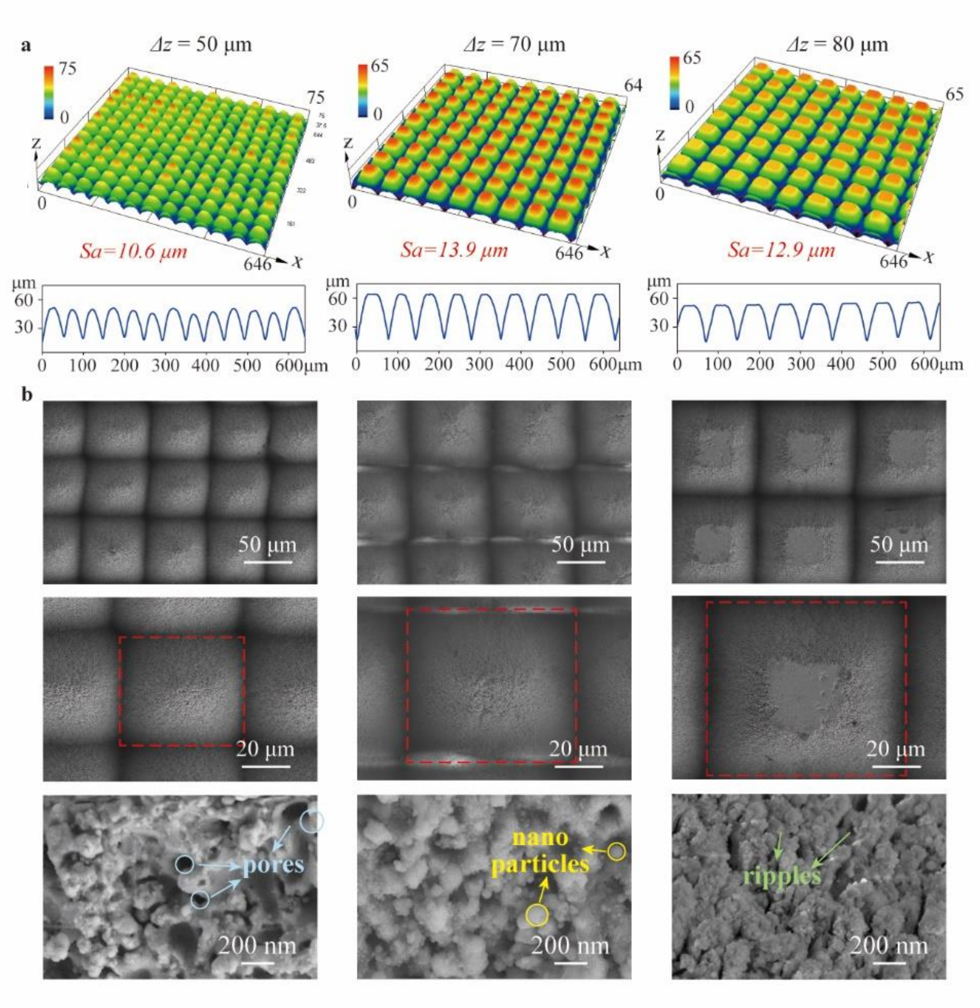

Figure S4. a) The 3D surface topographies of laser-ablated GYbZ bulk with different groove pitches (*Δz* = 50, 60, 70 and 80 μm). 2D profiles showing micro-morphology of laser-ablated GYbZ surfaces with different groove pitches (bottom). b) SEM images of laser laser-ablated GYbZ surfaces at different magnifications.

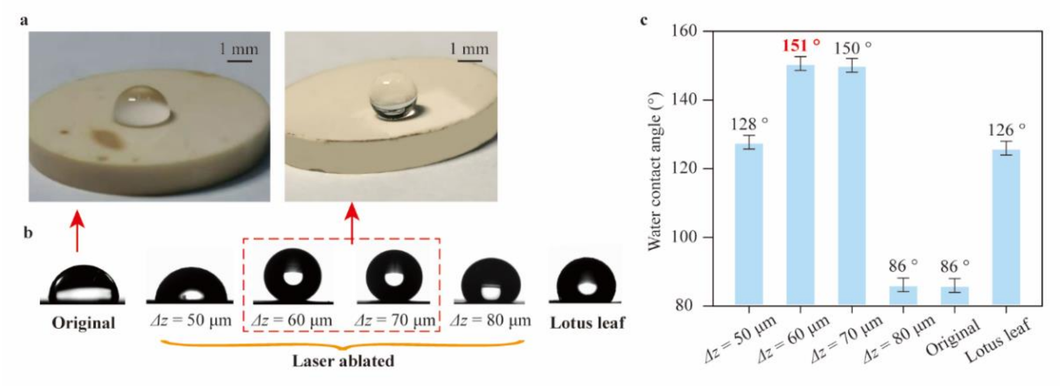

Figure S5. Room temperature wettability of as-prepared GYbZ bulks and lotus leaf surfaces. a) The wetting behavior of water droplets of 5 μl on the surface of the original GYbZ bulk (left), laser-ablated surface with Δz = 60 μm (right). (b) Images and (c) mean values of the contact angles of 5 μl water droplets on the surface of original, as-ablated bulks with different groove pitches and lotus leaf.

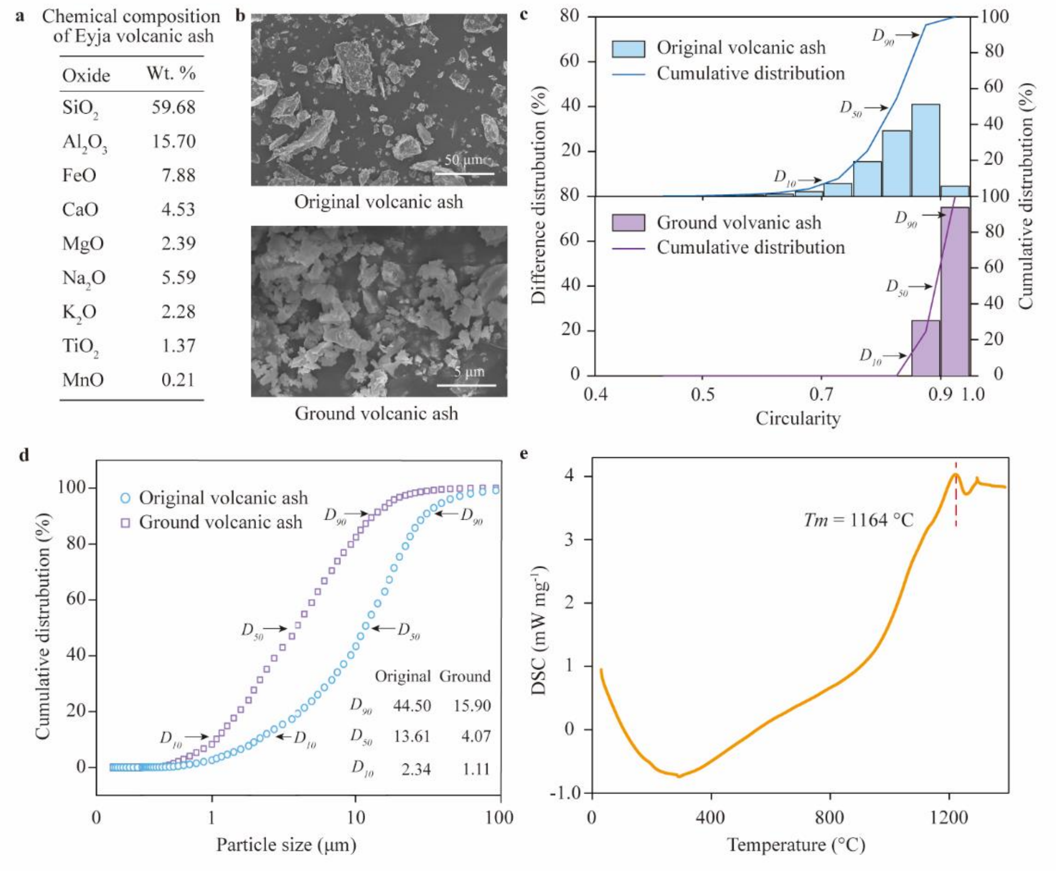

Figure S6. Characteristics of volcanic ash collected from 2010 eruption of Eyjafjallaj€okull volcano. a) Chemical composition of volcanic ash sample. b) SEM images of original (top) and ground volcanic ash (bottom). c) Difference and cumulative distribution of circularity of original (top) and ground volcanic ash (bottom). d) Cumulative particle size distribution of original and ground volcanic ash. The D_{10}, D_{50} and D_{90} define the particle size. e) DSC curve of the volcanic ash sample in oxidizing atmospheric conditions under a heating rate of 10 °C· min^{-1}, indicating the melting temperature T_m. A dashed line indicates the melting temperature, Tm.

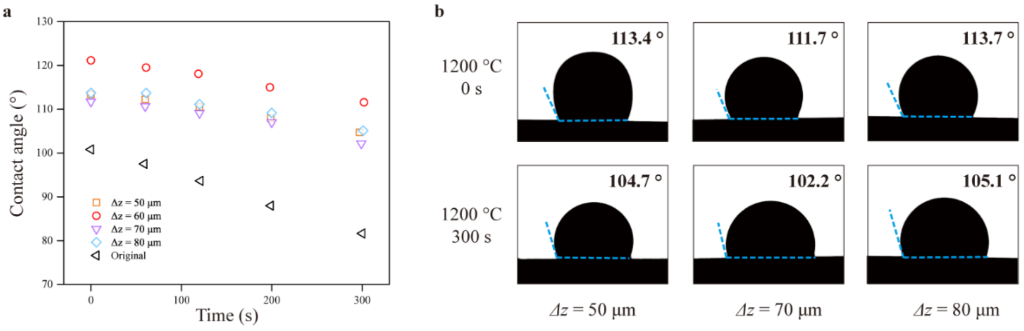

Figure S7. a) Relationships between contact angles of molten silicate on laser-ablated and original bulks and time. c) In-situ observations of volcanic ash on laser-ablated surfaces with different groove pitches (*Δz* = 50 μm, 70 μm and 80 μm) at 1200 °C.

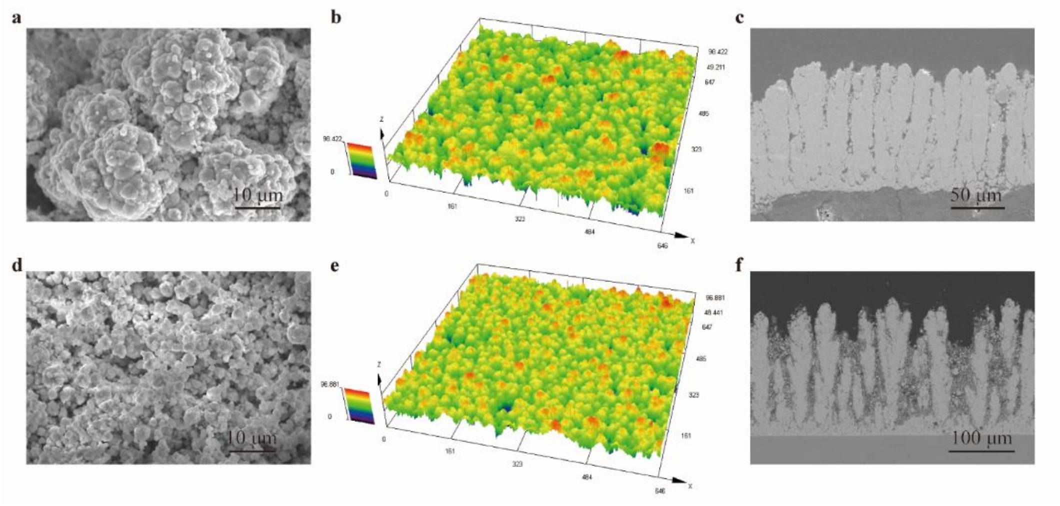

Figure S8. a) Surface images of PS-PVD GYbZ coatings sprayed at 800 mm (a) and 1200 mm (d). 3D surface topographies of PS-PVD coatings sprayed at 800 mm (b) and 1200 mm (e). Cross-sectional images of PS-PVD coatings sprayed at 800 (c) mm and 1200 mm (f).

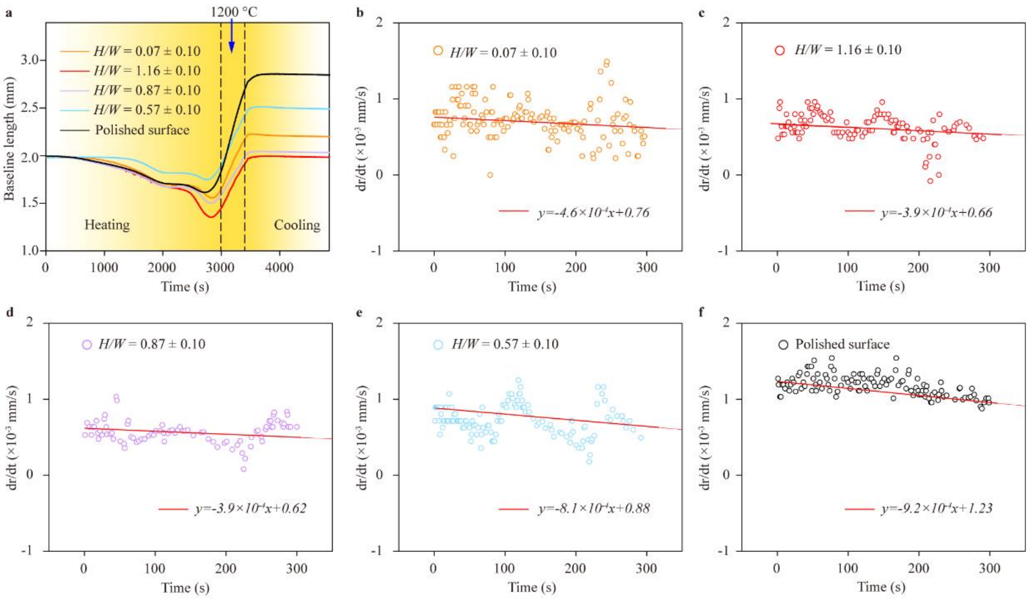

Figure S9. Spreading dynamics of molten volcanic ash droplets on various GYbZ surfaces. a) Evolution of the relative baseline length plotted as a function of relative time for molten volcanic ash droplets. b-f) Instantaneous spreading rate, dr/dt vs. time curves of GYbZ surfaces with different aspect ratio (H/W) and Polished GYbZ surface at 1200 °C, respectively.

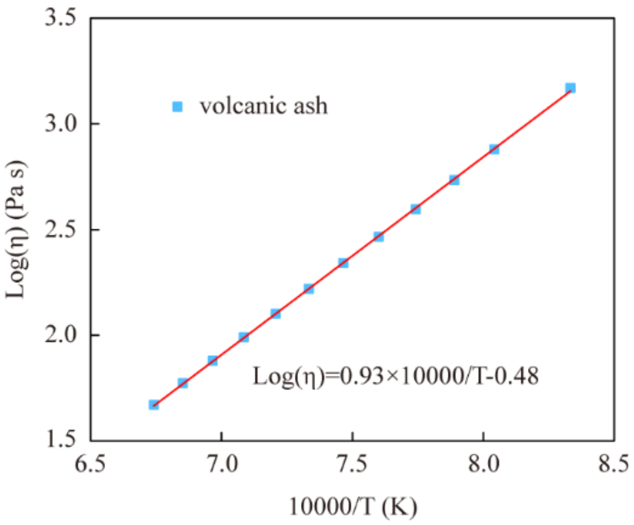

Figure S10. Viscosity of volcanic ash, η, as the function of temperature, T. The red line indicates the best-fit linear regression.

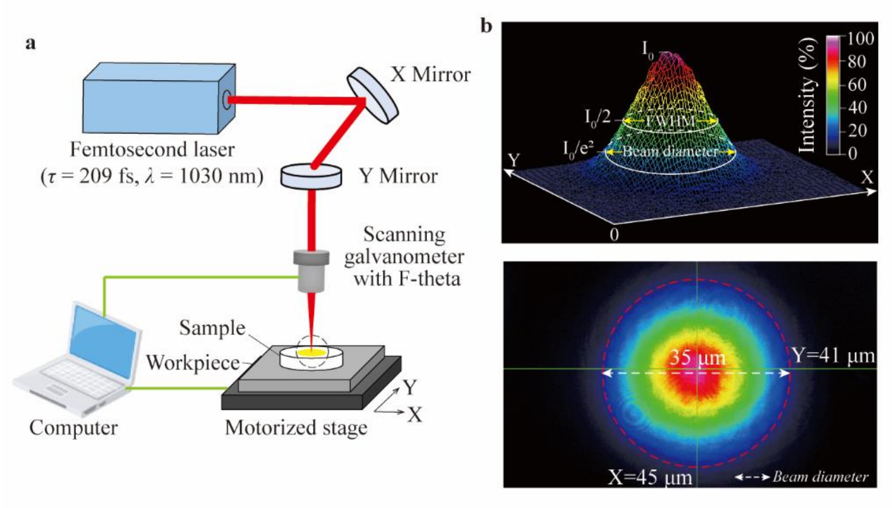

Figure S11. a) Experimental setup for ultrafast laser direct writing processing. Femtosecond laser beam is delivered and focused on GYbZ bulks via objective F-Theta scanning lens. b) The spatial intensity profile (left) showing the position of FWHM and beam diameter ($1/e^2$) and the facial intensity profile (right) showing the specific value of beam diameter, ~35μm.

2. Supplementary Table

Table S1. PS-PVD processing parameters for GYbZ coatings

	Net power (kW)	Current (A)	Ar (slpm)	He (slpm)	Feed rate (g/min)	Spray distance (mm)
1	60	2000	30	60	~4	800
2	60	2000	30	60	~4	1000
3	60	2000	30	60	~4	1200
